# GC-rich repeat expansions: associated disorders and mechanisms

**DOI:** 10.1515/medgen-2021-2099

**Published:** 2022-01-12

**Authors:** Christopher Schröder, Bernhard Horsthemke, Christel Depienne

**Affiliations:** Institute of Human Genetics, University Hospital Essen, University of Duisburg-Essen, Essen, Germany

**Keywords:** tandem repeat, repeat expansion, GC-rich repeats, regulatory regions, DNA methylation, histone modifications, RNA toxicity, RNA foci, nuclear inclusion, aggregation, RAN translation, RNA structure, monogenic disorders, long-read sequencing

## Abstract

Noncoding repeat expansions are a well-known cause of genetic disorders mainly affecting the central nervous system. Missed by most standard technologies used in routine diagnosis, pathogenic noncoding repeat expansions have to be searched for using specific techniques such as repeat-primed PCR or specific bioinformatics tools applied to genome data, such as ExpansionHunter. In this review, we focus on GC-rich repeat expansions, which represent at least one third of all noncoding repeat expansions described so far. GC-rich expansions are mainly located in regulatory regions (promoter, 5′ untranslated region, first intron) of genes and can lead to either a toxic gain-of-function mediated by RNA toxicity and/or repeat-associated non-AUG (RAN) translation, or a loss-of-function of the associated gene, depending on their size and their methylation status. We herein review the clinical and molecular characteristics of disorders associated with these difficult-to-detect expansions.

The human genome is particularly enriched in repetitions of adjacent nucleotide motifs, called tandem repeats [1], [2]. This dynamic class of variation has the highest mutational rate and is consequently highly polymorphic within human populations. The instability of tandem repeats increases in a length-dependent manner and their expansion across generations is a well-known process resulting in at least 50 human monogenic disorders [3], [4], [5]. Among those, at least one third are GC-rich. Here, we aim to review the disorders and mechanisms associated with GC-rich repeat expansions, focusing mainly on well-established monogenic conditions.

## Fragile X syndrome and associated disorders caused by CGG expansions in *FMR1*

**Figure 1:**
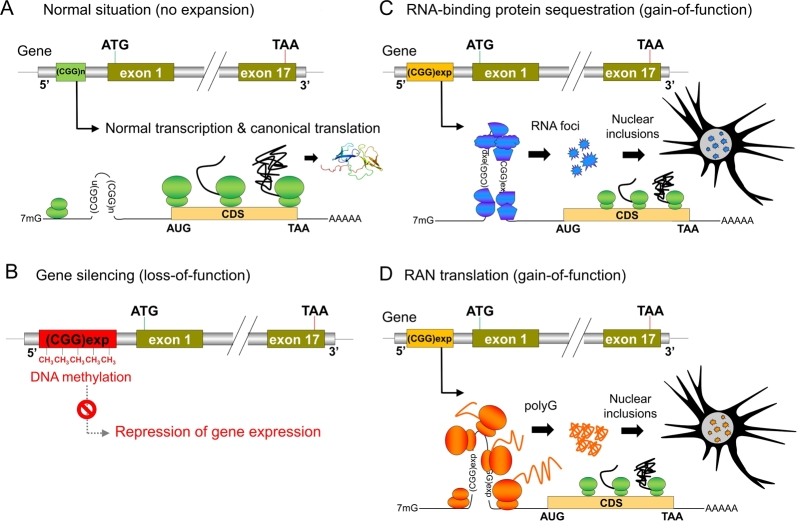
Main pathogenic mechanisms associated with GC-rich repeat expansions. (A) Nonpathogenic situation (e. g., less than 50 GCC repeats in *FMR1*) associated with normal transcription and canonical translation. (B) Epigenetic gene silencing. Full-length GC-rich expansions in gene promoters and/or 5′ untranslated regions (e. g., >200 CGG repeats in 5′UTR of *FMR1* causing Fragile X syndrome) are associated with DNA methylation at CpG sites. Expanded methylated alleles are locked in a chromatin configuration preventing gene transcription and protein expression. (C) Sequestration of RNA-binding splicing factors. Intermediate CGG expansions (55 to 200 repeats) causing Fragile X-associated tremor ataxia syndrome (FXTAS) can form stable RNA secondary structures able to bind specific RNA-binding proteins with high affinity. These RNA molecules accumulate to form inclusions in the nucleus and sequester bound RNA-binding proteins. (D) Repeat-associated non-AUG (RAN) translation is a noncanonical protein synthesis process in which peptide synthesis is initiated at the site of the expanded repeats in absence of an AUG codon. In the case of FXTAS, RAN translation leads to the synthesis of toxic polyglycine peptides that accumulate and form protein aggregates. Gain-of-function mechanisms described in (C) and (D) are mutually nonexclusive and can occur at the same time.

The first noncoding GC-rich expansion disorder, described in 1991, was Fragile X syndrome (FXS, MIM #300624) [6], [7]. FXS is one of the most frequent causes of intellectual disability (ID) and/or autism spectrum disorder (ASD) in males and is caused by CGG repeat expansions exceeding 200 repeats (full expansion) in the 5′ untranslated region (UTR) of the *FMR1* (FMRP translational regulator 1) gene (MIM #309550) on chromosome X. Above this threshold, CpGs contained within the CGG repeats are usually methylated and associated with an absence of *FMR1* expression [8], [9] (Figure 1A–B). Although point mutations leading to a loss-of-function of FMRP, the protein encoded by *FMR1*, are very rare, they can also cause FXS, confirming that loss-of-function of *FMR1* is the pathophysiological mechanism associated with full expansion [10]. Females with full *FMR1* expansion can also be affected depending on the X inactivation status of the mutated allele in the brain, but they usually present with milder symptoms compared to male individuals [11], [12].

Remarkably, CGG repeat expansions in *FMR1* ranging from 55 to 200 repeats (premutations) were later found to be associated with two other disorders: Fragile X-associated premature ovarian insufficiency (FXPOI, also known as Premature Ovarian Failure 1 [POF1], MIM #311360) in females [13] and Fragile X-associated tremor ataxia syndrome (FXTAS, MIM #300623) in males [14]. FXTAS is a neurodegenerative disorder mainly affecting males over 50 years of age. Ovarian insufficiency (FXPOI) occurs in 20–25 % of female *FMR1* premutation carriers and consists in absent or irregular cycles, lower fertility or infertility, and premature ovarian failure (i. e., complete cessation of menstrual periods before age 40). Contrary to full expansions, premutations are not associated with hypermethylation and do not prevent *FMR1* transcription and FMRP expression [15]. Noncoding expansions in *FMR1* have hence become a paradigm, illustrating how expansions in a single gene may have different downstream impacts and cause different disorders depending on their size [16].

Two major mechanisms have been proposed to explain FXTAS/FXPOI pathogenesis. One is a gain of function at the RNA level: unmethylated intermediate size CGG repeats can form stable secondary structures called G-quadruplexes and bind specific RNA-binding proteins, such as hnRNP A2B1, DROSHA, SAM68, and TDP-43 [17], [18], [19]. Aberrant RNA–protein complexes form RNA foci (also called inclusion bodies) and sequester bound proteins, preventing them from performing their normal function [20], [21], [22] (Figure 1C). The second pathogenic mechanism is the expression of toxic polypeptides directly produced by expansion by a process known as repeat-associated non-AUG (RAN) translation [23], [24], [25], [26]. RAN translation is a noncanonical protein synthesis process first described in spinocerebellar ataxia type 8 (SCA8, MIM #608768) and myotonic dystrophy type 1 (DM1, MIM #160900), in which peptide synthesis is initiated at the site of the expanded repeats in absence of an AUG codon [27] (Figure 1D). This process can theoretically occur in the three reading frames on both sense and antisense DNA strands, although only specific peptides are preferentially expressed or toxic. CGG repeats mainly lead to the abnormal expression of polyglycine (polyG) peptides that are also able to accumulate and form protein aggregates via a prion-like mechanism [24], [28], [29]. Although initially described in a pathological context, RAN translation could be a physiological process contributing to the regulation of *FMR1* expression in neurons by creating an upstream open reading frame (uORF) competing with FMRP expression [29].

RNA and protein gains-of-function are intimately linked together and probably both contribute to the pathogenesis of the disorder. Recent evidence shows that polyG peptides interact with pathogenic CGG repeat-derived RNA G-quadruplexes and that these RNA molecules could even promote the formation of polyG aggregates [28].

## GC-rich expansions associated with a loss-of-function

So far, only a few disorders other than FXS have been associated with GC-rich expansions causing epigenetic gene silencing (Table 1). Like FXS, CCG repeat expansions in the 5′UTR of *AFF2* (AF4/FMR2 family member 2, previously *FMR2*, MIM #300806) on chromosome X are associated with another X-linked ID disorder in males (FRAXE, MIM #309548) described in 1993 [30]. Intragenic deletions of *AFF2* have been identified in patients with ID [31], [32] and an excess of point mutations in *AFF2* has been described in males with ASD [33], further supporting the association of this gene with neurodevelopmental disorders.

Expansions associated with a loss-of-function have also been identified on autosomes. Most of the disorders described so far are recessive and, in this case, the disease is caused by an expansion in both alleles or by an expansion in one allele and a point variant in the other allele. The phenotype associated with expansions and point variants is usually identical. Such compound heterozygous alterations can be difficult to detect and their identification needs the combination of expansion detection and standard gene panel or exome analysis. At least three disorders corresponding to this description have been described so far.

Dodecamer (CCCCGCCCCGCG) expansions in the 5′UTR of *CSTB* (cystatin-B, MIM #601145) are responsible for progressive myoclonic epilepsy type 1 (EPM1, also known as Unverricht–Lundborg disease, MIM #254800), a recessive neurodegenerative epileptic condition characterized by tonic-clonic seizures and myoclonus [34]. Pathogenic *CSTB* expansions in both alleles or in one allele plus a point variant in the other allele cause the loss-of-function of cystatin B (stefin B), a small proteinase inhibitor, whose precise function still remains largely unknown [35].

More recently, CGG repeat expansions also leading to loss-of-function through hypermethylation have been described in *XYLT1* (xylosyltransferase 1, MIM #608124). Recessive variants in this gene had previously been described to cause Desbuquois dysplasia 2 (DBQD2, MIM #615777), a skeletal dysplasia associated with developmental delay, short stature, and facial characteristics. Expansions in *XYLT1* were uncovered using a combination of genome sequencing, microarray analysis, and Sanger sequencing in patients with Baratela-Scott syndrome (BSS), another skeletal dysplasia sharing many clinical features with DBQD2. The authors first identified homozygous or compound heterozygous pathogenic variants or deletions altering the coding region of *XYLT1* in a few patients. Segregation analysis of the variants within families revealed allelic drop-out, which prompted the authors to look for DNA methylation defects. This analysis revealed hypermethylation of alleles without point variants, consecutive to CGG expansions in the 5′UTR of *XYLT1* in a region that was incorrect in the reference genome [36]. BBS and DBQD2 are thus allelic disorders both linked to loss-of-function of *XYLT1* as a result of point variants or noncoding CGG expansions.

Finally, noncoding expansions in *GLS* (glutaminase, MIM #138280) in patients with global developmental delay, progressive ataxia, and elevated plasma glutamine (GDPAG, MIM #618412) were identified thanks to their associated biochemical phenotype. *GLS* encodes glutaminase, the enzyme catalyzing the first reaction of glutamine catabolism, an obvious candidate gene for elevated plasma glutamine. Heterozygous point variants in this gene were identified by exome sequencing in only two of three unrelated individuals with GDPAG, whereas all three had strongly impaired glutaminase activity, suggesting the existence of pathogenic variants undetected by exome sequencing. The authors then applied ExpansionHunter to genome sequence data and detected a GCA repeat expansion in the 5′UTR of *GLS* [37]. Expansions were either present in both alleles or in one allele, with a point variant in the other allele, and and exhibited a number of GCA repeats ranging from 400 to 1,500 (8–16 in control individuals).

Contrary to expansions causing FXS and BSS, expansions in neither *CSTB* nor *GLS* are hypermethylated and they both seem to lead to reduced gene transcription independently of DNA methylation [37], [38]. Unlike CGG expansions, GCA expansions in *GLS* do not contain any CpG, which is the only substrate of mammalian DNA methyltransferases, and thus cannot be methylated. Instead, they are associated with changes in histone modifications, including a decrease in transcriptionally active (H3K27ac and H3K4me3) marks and an increase in transcriptionally silent (H3K9me3) modifications [37]. These findings suggest a change in chromatin configuration as the result of the repeat expansion, as already shown for intronic GAA expansions in *FXN* (Frataxin, MIM #606829) causing Friedreich ataxia (FRDA, MIM #229300). These expansions alter the transcription of Frataxin by creating secondary DNA/RNA structures called R-loops, which block RNA polymerase and are associated with repressive histone marks [39], [40].

**Table 1:** List of noncoding repeat expansion disorders involving GC-rich (>66 % GC) motifs. BSS, Baratela-Scott syndrome; DM1, myotonic dystrophy type 1; DM2, myotonic dystrophy type 2; EPM1, progressive myoclonus epilepsy type 1 (Unverricht–Lundborg disease); FECD3, Fuchs endothelial corneal dystrophy type 3; FRAXE, Fragile XE syndrome; ALS/FTD, amyotrophic lateral sclerosis/frontotemporal dementia; FXS, Fragile X syndrome; FXTAS, Fragile X-associated tremor ataxia syndrome; GDPAG, global developmental delay, progressive ataxia, and elevated glutamine; NIID, neuronal intranuclear inclusion disease; OPDM1–3, Oculopharyngodistal myopathy type 1–3; OPML1, oculopharyngeal myopathy with leukoencephalopathy type 1; RCPS, Richieri-Costa–Pereira syndrome; SCA8/12/36, spinocerebellar ataxia type 8/12/36; AD, autosomal dominant; XL, X-linked; NA, not available; UTR, untranslated region.

Disorder	Inheritance	Chromosome	Gene	Location	Repeat motif	Normal repeat range	Pathological repeat range	Gene silencing (methylation)	RNA foci (sequestration)	RNA translation (toxic peptide)	References
NIID/OPDM3	AD	1q21.2	*NOTCH2NLC*	5′UTR/exon 1	CGG	7–40	≥60-500	variable (length-dependent)	yes (likely)	likely (polyG)	[41], [42], [43]
FRA2A	AD	2q11.2	*AFF3*	Intron	CGG	8–17	≥300	yes (yes)	NA	NA	[44]
GDPAG	AR	2q32.2	*GLS*	5′UTR	GCA	8–16	≥680-1,400	yes (no)	NA	NA	[37]
DM2	AD	3q21.3	*CNBP*	Intron	CCTG/CAGG	11–30	>50 -11,000	no	yes (yes)	yes (tetrapeptides)	[45]
SCA12	AD	5q32	*PPP2R2B*	5′UTR	CAG	4–32	≥43–78	no	yes (yes)	yes (polyQ, polyS)	[46]
OPDM1	AD	8q22.3	*LRP12*	5′UTR	CGG	13–45	90-130	no	likely	likely (polyG)	[41]
ALS/FTD	AD	9p21.2	*C9ORF72*	5′UTR/intron	GGGGCC	3–25	>30	variable (length-dependent)	yes (yes)	yes (dipeptides)	[47], [48]
OPML1	AD	10q22.3	*LOC642361*/*NUTM2B-AS1*	Noncoding transcript gene	CGG/CCG	3–16	40-60	NA	yes (likely)	likely (polyG)	[41]
FRA12A	AD	12q13.12	*DIP2B*	5′UTR	CGG	NA	NA	yes (yes)	NA	NA	[49]
SCA8	AD	13q21	*ATXN8/ATXN8OS*	3′UTR	CAG/CTG	15–50	>74–250	no	yes (yes)	likely (polyQ)	[50]
BSS	AR	16p12.3	*XYLT1*	Promoter	CGG	9-20	120-800	yes (yes)	NA	NA	[36]
RCPS	AR	17q25.3	*EIF4A3*	5′UTR	18- or 20-nucleotide motifs	3-12	15-16	NA	NA	NA	[51]
FECD3	AD	18q21.2	*TCF4*	Intron	CTG	5–31	>50	no	yes (yes)	NA	[52], [53]
OPDM2	AD	19p13.12	*GIPC1*	5′UTR	CGG	12–32	≥97-120	NA	likely	likely (polyG)	[54], [55]
DM1	AD	19q13.32	*DMPK*	3′UTR	CTG	5–37	>50–10,000	no	yes (yes)	likely (polyQ)	[56]
SCA36	AD	20p13	*NOP56*	Intron	GGCCTG	5–14	≥650-2,500	possible	yes (yes)	yes (dipeptides)	[57]
EPM1	AR	21q22.3	*CSTB*	promoter/5′UTR	CCCCGCCCCGCG	2–3	≥30-75	yes (no)	NA	NA	[34]
FXS	XL	Xq27.3	*FMR1*	5′UTR	CGG	5–50	>200	yes (yes)	no	no	[6], [7]
FXPOI	XL	Xq27.3	*FMR1*	5′UTR	CGG	5–50	55–200	no	yes (likely)	likely (polyG)	[13]
FXTAS	XL	Xq27.3	*FMR1*	5′UTR	CGG	5–50	55–200	no	yes (yes)	yes (polyG)	[14]
FRAXE	XL	Xq28	*AFF2*	5′UTR	CCG	4–39	≥200–900	yes (yes)	NA	NA	[30], [58]

So far, only very few disorders have been associated with dominant repeat expansion causing a loss-of-function: CGG expansions in *DIP2B* (Disco-interacting protein 2 homolog B, MIM #611379), associated with hypermethylation and a fragile site on chr12q13.12, have been reported to lead to a dominant nonsyndromic ID disorder (FRA12A, MIM #136630) [49]. However, no additional patients have been described since the initial study and this finding thus needs to be confirmed by additional reports. Likewise, CGG expansions in the 5′UTR of *AFF3* (AF4/FMR2 family member 3, MIM #601464) result in hypermethylation associated with the FRA2A fragile site [44]. Like *DIP2B* expansions, this disease–gene association also requires further evidence but point variants in *AFF3* have recently been associated with a dominant disorder including intellectual disability, mesomelic dysplasia, horseshoe kidney, and epileptic encephalopathy [59].

## GC-rich expansions associated with a gain-of-function

The first dominant noncoding repeat expansion disorders described were myotonic dystrophy type 1 (DM1) and spinocerebellar type 8 (SCA8), caused by CTG expansions in the 3′UTRs of *DMPK* (dystrophia myotonica protein kinase, MIM #605377) [56] and *ATXN8OS* (ATXN8 Opposite Strand LncRNA, MIM #603680) [50], respectively (Table 1). In the case of SCA8, a CAG expansion also exists on the reverse strand in the *ATXN8* coding gene (MIM #613289). The consequence of expansions in *DMPK* at the RNA level have extensively been studied. *DMPK* mRNA molecules containing CUG expanded repeats accumulate to form inclusions in muscle and neuron nuclei and sequester specific splicing factors such as the muscleblind-like 1 (MBNL1) protein. Consequently, the functional depletion of these RNA-binding proteins results in splicing defects of tissue-specific transcripts [60], [61]. RAN translation also occurs in DM1 and is possibly the main pathophysiological mechanism in SCA8, both mainly leading to the toxic expression of polyglutamine (polyQ) peptides, which are well known to adopt *β*-sheet structures prone to form insoluble fibrillar aggregates and neuronal intranuclear protein inclusions [27], [62]. RNA-dependent pathophysiological mechanisms and RAN translation of tetrapeptides (polyLPAC and polyQAGR) also coexist in myotonic dystrophy type 2 (DM2, MIM #602668), caused by CCTG/CAGG repeat expansions in *CNBP* (CCHC-type zinc finger nucleic acid-binding protein, MIM #116955). These polypeptides are able to accumulate specifically in neurons, astrocytes, and white matter structures and are toxic independently of RNA foci and nuclear sequestration of MBNL1 by CCUG transcripts. This suggests a pathophysiological model in which an RNA-dependent pathogenic mechanism first occurs in the nucleus and when sequestration capacity is exceeded, RNAs are exported to the cytoplasm where they undergo RAN translation [63].

Another example of these complexed intertwined mechanisms is exemplified by hexanucleotide (GGGGCC) expansions in *C9ORF72* (chromosome 9 open reading frame 72, MIM #614260). These expansions located in the 5′UTR (or first intron depending on the isoform) of *C9ORF72* cause a dominant disorder characterized by frontotemporal dementia, amyotrophic lateral sclerosis, or the association of both at the individual level and/or within families (ALS/FTD, MIM #105550). *C9ORF72* G_4_C_2_ repeats, like GCC repeats, are able to adopt G-quadruplex structures [64], and they can sequester multiple RNA-binding (mainly SRSF and hnRNP) proteins in a cell type-specific manner [65]. RAN translation of *C9ORF72* G_4_C_2_ repeats in sense and antisense produces five dipeptide proteins (polyGA, polyGP, polyGR, polyPA, polyPR), three of which (polyGR, polyPR, and polyGA) are highly toxic [66], [67], [68], [69], [70], [71], [72], [73]. Remarkably, polyPR and polyGA peptides are able to spread from one cell to the other via exosome-dependent but also exosome-independent mechanisms [74], [75]. Finally, *C9ORF72* G_4_C_2_ repeats can be methylated in a length-dependent manner and DNA methylation inversely correlates with repeat size and age at disease onset [76], [77], [78]. Loss-of-function of *C9ORF72* is insufficient to lead to ALS/FTD and no truncating mutations associated with ALS/FTD have been reported in this gene, but recent evidence suggests that *C9ORF72* haploinsufficiency could contribute to disease pathogenesis by worsening the repeat-dependent gain-of-function mechanisms [79]. The examples of DM1, SCA8, and *C9ORF72*-associated ALS/FTD show that mechanisms involving toxic RNA, RAN translation, and loss-of-function through hypermethylation are not mutually exclusive and can even have additive effects, each explaining parts of the pathophysiogenesis.

Recently, four additional dominant GC-rich repeat expansions have been identified (Table 1). The most frequent is a CGG repeat expansion in *NOTCH2NLC* (Notch 2 N-Terminal Like C, MIM #618025), one of four human-specific genes (*NOTCH2NLA*, *NOTCH2NLB*, *NOTCH2NLC*, and *NOTCH2NLR*) sharing a high degree (>99 %) of DNA homology and originating from pericentromeric tandem duplications of the 5′ part of *NOTCH2* on chromosome 1. These expansions, located in the 5′UTR/first exon of *NOTCH2NLC*, cause a dominant neurodegenerative disorder called neuronal intranuclear inclusion disease (NIID, MIM #603472). This condition is clinically variable and characterized by eosinophilic intranuclear inclusions in neurons, glial cells, fibroblasts, and muscles. The age at onset is also highly variable, ranging from infancy to late adulthood, although most patients show the first symptoms from the third decade of life. Clinical features include pyramidal and extrapyramidal symptoms, cerebellar ataxia, cognitive decline, peripheral neuropathy, and autonomic dysfunction [80]. Moreover, most patients typically show white matter abnormalities on brain MRI reminiscent of those occasionally observed in FXTAS, including T2-weighted hyperintensity signals in the middle cerebellar peduncles and high-intensity signals in the corticomedullary junction on diffusion-weighted imaging. *NOTCH2NLC* expansions were concomitantly described in three independent studies. Ishiura et al. used TRhist and long-read Single Molecule Real-Time (SMRT) sequencing to identify *NOTCH2NLC* expansions [41]. Sone et al. and Tian et al. first performed genome-wide linkage analysis to identify overlapping intervals on chromosomes 1p22.1-q21.3 and 1p13.3-23.1 before using SMRT and/or nanopore long-read sequencing to detect and characterize *NOTCH2NLC* expansions [42], [43]. Numerous follow-up studies revealed the presence of pathogenic *NOTCH2NLC* expansions in patients with essential tremor (ETM6, MIM #618866) [81], [82], FTD and Alzheimer-like dementias [43], [83], Parkinsonism [43], [84], [85], multiple system atrophy [86], and oculopharyngodistal myopathy (OPDM3) [87], [88]. *NOTCH2NLC* expansions are more frequent in Japan and China, due to a founder effect in Asian populations, but can also be present in patients from other geographic origins including Europe and can even occur *de novo* in sporadic cases [89]. Pathogenic expansions range from 60 to more than 500 repeats whereas control individuals have less than 40 CGG repeats [41], [42], [90], [91]. *NOTCH2NLC* is correctly annotated only in the hg38 reference genome and the diagnostic testing of the expansion is complicated by the almost identical *NOTCH2NL* copies, the GC-rich nature of the expansions as well as the presence of interrupting AGG motifs present in a subset of both healthy and affected individuals [90]. *NOTCH2NCL* expansions are not associated with DNA hypermethylation and do not consistently alter the expression of *NOTCH2NLC*, but antisense transcripts are specifically produced in affected individuals, suggesting pathological mechanisms involving a toxic gain of function at the RNA level and/or the existence of RAN translation [41], [42], [92]. A recent study confirmed that RNA molecules with expanded CGG repeats can form RNA foci and sequester RNA-binding proteins into p62-positive intranuclear inclusions specifically in affected individuals [91].

Similar CGG expansions in at least three different genes have been identified in oculopharyngeal myopathy (OPML) and oculopharyngodistal myopathy (OPDM). Two of these expansions both composed of CGG repeats in *LOC642361*, a long noncoding RNA gene overlapping the *NUTM2B-AS1* antisense transcript (NUTM2B Antisense RNA 1, MIM #618639) on Chr. 10q22.3, and in the 5′UTR of *LRP12* (low-density lipoprotein receptor-related protein 12, MIM #618299) were identified by Ishiura and collaborators using the same strategy that initially detected *NOTCH2NLC* expansions [41]. *LRP12* expansions were detected in several families with oculopharyngodistal myopathy (OPDM1, MIM #164310), a neuromuscular disorder in which muscular weakness in the legs and arms is associated with external ophthalmoplegia, dysphagia, and ptosis, while expansions in *LOC642361*/*NUTM2B-AS1* were found in a single family with oculopharyngeal myopathy, limb weakness, ataxia, ptosis, and white matter abnormalities similar to those seen in NIID (OPML1, MIM #618637). Two independent studies identified CGG repeat expansions in the 5′UTR of *GIPC1* (MIM #605072) in multiple families with OPDM2 (MIM #618940) using a combination of whole-genome sequencing and long-read sequencing [54], [55]. *GIPC1* expansions lead to increased mRNA expression but do not affect protein expression [54]. Altogether, these recent studies indicate that CGG expansions in multiple genes lead to dominant neurodegenerative disorders irrespectively of the gene where they occur by mechanisms that likely resemble those described in FXTAS and *C9ORF72*-associated FTD/ALS [92].

The relationship between GC-rich repeat expansions, DNA methylation, and gene expression remains unclear. Although *FMR1* expansions have outlined a clear correlation between expansion size and hypermethylation locking the gene in an unexpressed state, the same threshold does not seem to apply to all genes equally. Indeed, expansions containing more than 200 GCC repeats in *NOTCH2NLC* or *C9ORF72* for instance are not consistently associated with DNA methylation. Contrary to full expansions in *FMR1*, large expansions in these genes still allow transcription of RNA molecules containing expanded repeats and RAN translation of toxic polypeptides able to aggregate and form inclusions. DNA methylation could even be protective in some *NOTCH2NLC*-associated NIID [91] while worsening the effect of RNA and peptide toxicity in *C9ORF72*-associated ALS/FTD [79]. In this setting, studying how DNA methylation impacts the progression of each disorder associated with GC-rich expansions is of crucial importance as this information could be used to design new treatment strategies based on Cas9 methylation editing, as recently suggested for FXS [93].

## Perspectives on the identification of GC-rich expansions

Most recent studies reporting repeat expansions relied on large and/or multiple families that allowed the identification of a genomic interval prior to the expansion. Some of the recently identified disorders turned out to occur quite frequently, suggesting that many rarer disorders associated with undetected repeat expansions exist. Indeed, because of their repetitive nature, high degree of polymorphism, and abundance in human genomes, repeat expansions remain difficult to detect by standard amplification or sequencing technologies. Repeat expansions can be looked for from short-read sequencing data using specific tools such as LobSTR [94], HipSTR [95], TREDPARSE [96], ExpansionHunter [97], STRetch [98], GangSTR [99], and exSTRa [100], but most of these tools need inputs regarding the genomic region and repeat motifs and their use is often limited to detect already known expansions. So far, only two bioinformatics tools, TRhist [101] and ExpansionHunter DeNovo [102], can detect any type of repeat expansions at a genome-wide scale. However, since short reads encompassing repeats usually map to multiple genomic regions and are clipped off or discarded, the existing tools tend to perform poorly in estimating the number of repeats on each allele and underestimate the number of repeats, especially in the case of very large expansions. For this reason, long-read technologies including Oxford Nanopore sequencing and SMRT sequencing have become the new standard to detect and characterize repeat expansions [103]. These powerful technologies detect several hundreds of structural variants per individual that mostly correspond to polymorphic repeat elements. The normal repeat ranges and associated allele frequencies of these repeat variations have not yet been described in large control populations, and the detection of expansions without hypothesis on the expanded motif or genomic region where it is located thus remains a challenge in practice. However, this field is rapidly evolving. Specific tools like NanoSatellite [104] and tandem-genotypes [105] have already been developed to specifically study repeats present in long-read data and we can hope that the identification of repeat expansions will be soon integrated to standard genetic pipelines.

Although GC-rich repeat expansions are mainly known to cause monogenic disorders, it is very likely that this type of genetic variation also largely contributes to the genetic architecture of complex disorders [106]. A recent study investigating the contribution of tandem repeats to the risk of developing ASD in cohorts of >5,000 patients showed that repeat expansions were more prevalent in subjects with ASD (23.3 %) than their healthy siblings (20.67 %). These findings suggest that repeat expansions at more than 2,500 loci account for 2.6 % of autism risk [107]. Most of the top candidate regions identified are GC-rich and include known repeat expansion loci (*FMR1*, *FXN*, and *DMPK*) as well as new loci in genes associated with monogenic disorders, such as *MBOAT7*, *CDON*, *IL1RAPL1*, and *FGF14*. Another study focusing on *de novo* repeat changes showed a significant excess of repeat expansion in ASD subjects. Interestingly, these *de novo* repeat additions mainly occur in conserved fetal brain regulatory regions [108]. Repeat expansions located in regulatory regions, which are very often GC-rich, have been shown to have a significant impact on gene expression [109], [110]. This observation holds true for copy number variation involving microsatellites (i. e., motifs less than 1–9 bp), but for variable number of tandem repeats (VNTR), involved motifs are ≥10 bp [111]. Further studies are therefore required to address this complexity and clarify the role of tandem repeat expansions in rare and more common human disorders.
